# Identifying models of trait‐mediated community assembly using random forests and approximate Bayesian computation

**DOI:** 10.1002/ece3.5773

**Published:** 2019-11-21

**Authors:** Megan Ruffley, Katie Peterson, Bob Week, David C. Tank, Luke J. Harmon

**Affiliations:** ^1^ Department of Biological Sciences University of Idaho Moscow ID USA; ^2^ Institute for Bioinformatics and Evolutionary Studies (IBEST) Moscow ID USA; ^3^ Stillinger Herbarium University of Idaho Moscow ID USA

**Keywords:** approximate Bayesian computation, community assembly, competitive exclusion, environmental filtering, random forest

## Abstract

Ecologists often use dispersion metrics and statistical hypothesis testing to infer processes of community formation such as environmental filtering, competitive exclusion, and neutral species assembly. These metrics have limited power in inferring assembly models because they rely on often‐violated assumptions. Here, we adapt a model of phenotypic similarity and repulsion to simulate the process of community assembly via environmental filtering and competitive exclusion, all while parameterizing the strength of the respective ecological processes. We then use random forests and approximate Bayesian computation to distinguish between these models given the simulated data. We find that our approach is more accurate than using dispersion metrics and accounts for uncertainty in model selection. We also demonstrate that the parameter determining the strength of the assembly processes can be accurately estimated. This approach is available in the R package CAMI; Community Assembly Model Inference. We demonstrate the effectiveness of CAMI using an example of plant communities living on lava flow islands.

## Graphical abstract

Using an adapted model of phenotypic similarity and repulsion, we are able to simulate the process of community assembly via environmental filtering and competitive exclusion, all while parameterizing the strength of the respective assembly processes. We then use approximate model selection approaches to distinguish between assembly models and estimate the strength of non‐neutral assembly processes.

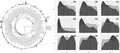

## 1 INTRODUCTION

Though methods to infer community assembly vary, many approaches share a central idea based on phylogenetics; the pattern of shared evolutionary history between species that coexist provides insight into the historical processes that assembled the community (Brooks & McLennan, 1991; Grandcolas, 1998; Losos, 1996; Thompson et al., 2001; Webb, 2000; Webb, Ackerly, McPeek, & Donoghue, 2002). To gain insight into the assembly process, a collection of metrics have been used to characterize the patterns of diversity in a community using species/genus ratios and other higher taxonomic diversity metrics (Faith, 1992; Gotelli & Colwell, 2001; Magurran, 1988; Weiher & Keddy, 1995). Though informative, these patterns often provide little information about the processes that generated them (Peters, 1991). Functional traits provide information about diversity and niche space within a community (Macarthur & Levins, 1967; McGill, Enquist, Weiher, & Westoby, 2006; Weiher et al., 1999) and have long been used to understand resource partitioning between species, as well as coexistence (Cornwell, Schwilk, & Ackerly, 2006; de Bello et al., 2009; Kraft, Cornwell, Webb, & Ackerly, 2007; Kraft, Godoy, & Levine, 2015). Though the collection and dimensionality of trait data is at times insurmountable, turning to phylogenetic information as a proxy for functional traits was, and is, a viable alternative. Measures of phylogenetic diversity and dispersion, which carry more information than higher taxonomic categories and hopefully, encompass trait information, have become widely used in community ecology to infer community assembly processes (Cavender‐Bares, Keen, & Miles, 2006; Kembel et al., 2010; Miller, Farine, & Trisos, 2017; Webb, 2000; Webb, Ackerly, & Kembel, 2008; Webb et al., 2002). These metrics focus on identifying alternative models of community assembly, environmental filtering and competitive exclusion. Environmental filtering occurs when the abiotic properties of an environment physically keep a species from existing there (Bazzaz, 1991). Competitive exclusion describes when species that share the same or similar niche space compete for resources resulting in some species being excluded from the community altogether, also referred to as limiting similarity (Macarthur & Levins, 1967). To determine whether non‐neutral processes have predominantly influenced assembly patterns, phylogenetic dispersion metrics, such as mean pairwise distance (MPD) and mean nearest taxon distance (MNTD)—which can be calculated using phylogenetic branch lengths, number of nodal distances, and phenotypic distances—are used to compare observed community dispersion to null expectations (Gotelli & Colwell, 2001; Kembel et al., 2010; Webb, 2000; Webb et al., 2008, 2002).

More specifically, inferences of the assembly process using dispersion metrics are determined in a statistical hypothesis testing framework using several randomly generated null models (Conner & Simberloff, 1979; Gotelli & Graves, 1996). Commonly, the standard effect size of dispersion metrics, known as net relatedness index (NRI) for MPD and nearest taxon index (NTI) for MNTD (Webb, 2000), is used as the test statistic to measure significance of the observed community dispersion compared to null expectations of community dispersion if the community was assembled randomly. However, inference is conditional on the assumption that the relevant phenotypes for the environment or competition are phylogenetically conserved among the species in the community, or harbor strong phylogenetic signal within the community of focus. If this assumption is true, and environmental filtering has predominately impacted the assembly process, the phylogenetic data are expected to be significantly clustered, or under‐dispersed, in the local community. Likewise, when considering a community assembled by competitive exclusion, we expect to see significantly less shared evolutionary history as compared to null expectations or significant phylogenetic over‐dispersion (Cavender‐Bares et al., 2006; Webb, 2000; Weiher & Keddy, 1995).

The dubious assumption of strong phylogenetic signal between the phylogeny and phenotypes is a main critique of these approaches. Kraft et al. (2007) showed via simulations that when the assumption of phylogenetically conserved traits was even mildly violated, phylogenetic dispersion metrics were inadequate to infer community assembly processes. Furthermore, this violation of assumptions can, in fact, lead to patterns contrary to those expected for a given assembly process (Cavender‐Bares, Kozak, Fine, & Kembel, 2009; Gerhold, Cahill, Winter, Bartish, & Prinzing, 2015; HilleRisLambers, Adler, Harpole, Levine, & Mayfield, 2012; Mayfield & Levine, 2010; Weiher & Keddy, 1995, Weiher & Keddy, 1999). To circumvent this issue, one can assess whether or not functional traits of interest for the community are phylogenetically conserved, and then use that information to guide the inference procedure (Kembel et al., 2010; Kraft et al., 2007). Though, if functional trait information is available, it is typically used in consort with phylogenetic information because using phenotypic information alone relies on expectations for how the phenotypes should be distributed in the community to infer non‐neutral processes (de Bello et al., 2009; Graham, Parra, Tinoco, Stiles, & McGuire, 2012). While in many instances both phylogenetic dispersion and phenotypic dispersion are measured and analyzed in a similar framework (HilleRisLambers et al., 2012), an approach that integrates both to simultaneously estimate support for alternative assembly models is lacking.

Finally, the inference procedure using dispersion metrics relies on statistical hypothesis testing, and therefore, on how well the null model represents neutral expectations. Currently, there exists an extensive number of null models that can be used to infer assembly processes, ranging from simple null models based on random shuffling of taxon labels (Cornwell et al., 2006; Gotelli & Graves, 1996; Gotelli, 2000; Kembel et al., 2010; Webb et al., 2002), to incredibly dynamic null models (Pigot & Etienne, 2015) and analytical frameworks (Stegen et al., 2013) that incorporate macroevolutionary processes such as speciation, dispersal, and extinction. There also exist simulation software (Münkemüller & Gallien, 2015) to simulate the process of assembly with trait information mediating which species enter the community. However, even with more dynamic null models and simulation power, relying on statistical hypothesis testing and passing a significance threshold to infer an assembly process are problematic, in part due to the sensitivity between *p*‐values and sample size and how we interpret “significance,” but also because each analysis of a particular data type and test statistic results in a measure of significance. Researchers are then responsible for integrating across a suit of hypothesis tests, some that may be significant while others are not, in order to draw an inference. Arguably, a model‐based inference procedure is necessary to incorporate all data at once, rank models of community assembly by their relative support, and, importantly, incorporate uncertainty in model inference. In this model‐based inference procedure, we can simultaneously weigh the support for each community assembly model while also considering both phylogenetic and phenotypic data in the regional and local community. When each model garners a portion of support given the data, we are able to understand when a dominant signal of non‐neutral or neutral assembly is present in the data (i.e., strong support for one model), when two processes are acting simultaneously (i.e., strong support for two models), and when the data lack signal to identify a dominant process (i.e., equal support across all models).

Several approaches have implemented model‐based inference procedures for community assembly already (Munoz et al., 2018; Pontarp, Brännström, & Petchey, 2019; van der Plas et al., 2015), paving the way to measuring the relative impact of different processes on community assembly. However, we still lack a method that integrates both phylogenetic and phenotypic information in a species‐based model where the strength of the non‐neutral processes can be estimated. Here, we develop a stochastic algorithm to simulate communities assembled under environmental filtering and competitive exclusion processes by adapting coevolutionary phenotypic matching and repulsion models. In doing this, we avoid having to make any assumptions about how the traits have evolved along the phylogeny. Our approach simultaneously considers the phylogenetic and phenotypic information from species in the local and regional communities and parameterizes the relative strength of the assembly processes realizing strong to mild non‐neutral assembly. Finally, we implement a model selection inference procedure by using two approximate approaches, random forests (RF; Breiman, 2001; Breiman & Cutler, 2007) and approximate Bayesian computation (ABC; Csilléry, Blum, Gaggiotti, & François, 2010). We acknowledge that while these assembly processes are often happening simultaneously in nature, when investigating a targeted trait hypothesized to play a role in the non‐neutral assembly of a particular community, the model selection inference procedure holds power to detect the most conspicuous process, if applicable. We are using both model selection approaches because, though RF has been used for model selection in other contexts, it has not been used to distinguish between community assembly models like ABC has (Munoz et al., 2018; Pontarp et al., 2019; van der Plas et al., 2015); thus, we document a comparison and collaboration of the two approaches here.

We make our approach available as an R package, CAMI, Community Assembly Model Inference (https://github.com/ruffleymr/CAMI). To demonstrate the effectiveness of CAMI, we use power analyses to show that our approach more accurately infers models of community assembly compared to hypothesis testing using dispersion metrics. We also show that the parameter governing the strength of the assembly processes can be accurately estimated using ABC. Finally, we demonstrate community assembly model inference and parameter estimation using CAMI with an empirical example from the plant communities that exist on lava flow islands in Craters of the Moon National Monument and Preserve.

## 2 METHODS

### 2.1 Community assembly models

We focus on three community assembly models: neutral, environmental filtering, and competitive exclusion. For all models, we assume communities are assembled from a regional pool of species where each species in the regional pool is equally likely to colonize the local community. We also assume the phylogenetic relationship between all species is known and that there is continuous trait information for all species. We simulate the assembly of a local community from the regional species pool under one of the three models. Under the neutral model of assembly, all species in the regional community have an equal probability of persisting in the local community (Hubbell, 2001; Rosindell, Hubbell, He, Harmon, & Etienne, 2012). The probability that a given species survives, or persists, in a non‐neutrally assembled community is not equal for all species, and these varying probabilities of persistence drive the alternative models of community assembly.

To model environmental filtering, we adapt an approach from coevolutionary models (Nuismer & Harmon, 2015; Nuismer, Jordano, & Bascompte, 2013) to relate trait interactions between species and their environment with the probability of surviving in a community. For interactions between species and their environment, we implement a phenotypic matching mechanism where the probability, $P\left(z_{i},z_{E}\right)$, of a species persisting in the local community increases when the phenotype of the species $z_{i}$ and the optimal phenotype of the environment $z_{E}$ are more similar:

(1) $$P\left(z_{i},z_{E}\right)=Exp\left[-\frac{1}{t_{E}}{\left(z_{i}-z_{E}\right)}^{2}\right]$$

The probability a species with phenotype, $z_{i}$, persists in an environment with a phenotypic optimum, $z_{E}$, also depends on the strength of the environmental filtering, $t_{E}$. When $t_{E}$ is large, filtering has a mild effect in that species are less penalized for having phenotypes dissimilar to the environmental optimum, whereas when $t_{E}$ is small, the filtering effect is stronger because species are heavily penalized for phenotypes dissimilar to the optimum.

To model competitive exclusion, the probability, $P\left(z_{i},\bar{z}\right)$, of a species persisting in the local community increases as the phenotype of the species $z_{i}$ and the mean phenotype of the local community $\bar{z}$ are more dissimilar.

(2) $$P\left(z_{i},\bar{z}\right)=1-Exp\left[-\frac{1}{t_{C}}{\left(z_{i}-\bar{z}\right)}^{2}\right]$$

Here, the probability a species with phenotype, $z_{i}$, persists in a community with mean phenotypic, $\bar{z}$, depends on the strength of competition between species, $t_{C}$. When $t_{C}$ is large, competition has a strong effect in that species are heavily penalized for having phenotypes similar to the mean phenotype of the local community. When $t_{C}$ is small, competition is weaker in that species are less penalized for having a phenotype similar to the mean phenotype of the community.

### 2.2 Data simulation

**Figure 1:**
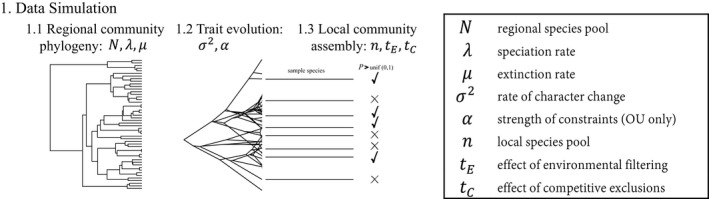
Outline of data simulation process. (1.1) Simulate the regional phylogeny. (1.2) Simulate trait evolution along the regional phylogeny. (1.3) Simulate the assembly of the local community by sampling species at random from the regional species pool and calculating the probability of persistence for each sampled species. These probabilities are calculated differently depending on the model of assembly being simulated, and if a species' probability of persistence is greater than a randomly generated probability, then that species survives in the local community

For a single simulation of community assembly, first, a regional community phylogeny is simulated under a constant birth–death process with speciation, *λ*, and extinction, *μ*, parameters, until the desired number of regional species, *N*, is reached (Figure 1; Stadler, 2011). Traits are evolved on the regional phylogeny, one for each species, (Revell, 2012) under either a Brownian Motion (BM; Felsenstein, 1985) or Ornstein‐Uhlenbeck (OU) model of trait evolution (Butler & King, 2004; Hansen, 1997) characterized by the rate of character change, $\sigma^{2}$, and, for OU models, the “strength of pull” to the trait optimum, *α* (Figure 1). Traits evolve under BM in a way that mimics drift over macroevolutionary timescales and OU does the same only it includes a selective regime in which traits are “pulled” toward a phenotypic optimum. We simulate under these different models of trait evolution because they do not enforce the assumption that trait differences are correlated to phylogenic differences and create more variability in how the data behave under the assembly models. Once the regional community exists with phylogenetic relationships and trait information, the assembly of the local community can begin.

The assembly process uses the probabilities of species persisting in local communities, $P\left(z_{i},z_{E}\right)$ for environmental filtering and $P\left(z_{i},\bar{z}\right)$ for competitive exclusion, and a rejection algorithm to stochastically assemble the local community. When simulating under a competition model, the strength of competition between species, $t_{C}$, parameterizes the assembly process. Likewise, under an environmental filtering model, the strength of the environmental filter, $t_{E}$, along with the environmental phenotypic optimum, $z_{E}$, parameterizes the assembly process. For the investigative simulations, the phenotypic optimum is determined by a random draw from the simulated trait distribution of the regional community, and it remains constant throughout an entire simulation.

When a species colonizes the community, the probability of persistence is calculated, and the species is included in the local community if that probability is greater than a uniform random number between 0 and 1 (Figure 1). Otherwise, the species is rejected from being in the local community. This stochasticity included in the algorithm is more apparent in the emergent data when the ecological strength parameter is imposing weak non‐neutral assembly. When a species is rejected from entering the community, it remains in the regional pool and is still able to colonize the local community again. In this case, the probability of persistence is recalculated, and the species has another chance to pass the rejection algorithm. As in the neutral model, the assembly process ends when the local community has reached species richness capacity, n.

All parameters mentioned are either fixed or drawn from a prior distribution. Information regarding the default prior distributions and fixed values for each parameter can be found in Table S1 or in the help documentation for the R package “CAMI” (https://github.com/ruffleymr/CAMI). Any parameter mentioned, along with prior distributions, can also be set by the user. In simulations described here, the default prior distributions were used unless otherwise stated.

### 2.3 Inference procedure

For a single simulation of community assembly, a regional and local phylogeny and a regional and local distribution of trait values is returned. This information is summarized in 30 different summary statistics that capture information about the phylogeny, trait distributions, and phylogenetic signal within the traits of the local community (Garland, Harvey, & Ives, 1992; Purvis & Rambaut, 1995; Deevi, 2016; Janzen, Höhna, & Etienne, 2015; Kendall, Boyd, & Colijn, 2018; Komsta & Novomestky, 2015; Paradis & Schliep, 2018; Pennell, FitzJohn, Cornwell, & Harmon, 2015; Table S2). These summary statistics are then used for model selection and parameter estimation.

To predict model probabilities from empirical data, we used two model selection approaches. The first approach uses a machine learning classification algorithm, random forests (RF; Breiman, 2001; Liaw & Wiener, 2002), to build a “forest” of classification trees using the simulated summary statistics as predictor variables and the community assembly models as response variables. As a classifier is being built, RF is simultaneously measuring the “Out of Bag” (OoB) error rates of the classifier by cross‐validating each classification tree with a subset of the original data that was not used to make the tree in question. The OoB error rates measure how often the data are incorrectly classified. Additionally, RF quantifies the effect of including each summary statistic on the accuracy of the classifier through two variable importance measures, mean decrease in accuracy (MDA) and Mean decrease in Gini Index (GINI) (Breiman, 2002).

Random forests is generally robust to noisy and/or overpowering predictor variables because each tree in the forest is constructed with a random subset of the data and predictor variables (Breiman & Cutler, 2007), which reduces the correlation among the trees while still improving the overall predictive power of the forest. The second approach, ABC, when using the rejection algorithm, relies on the Euclidean distance between observed and simulated summary statistics to accept simulations into the posterior probability distribution of the models given the data (Csilléry et al., 2010). The support for each model then comes from the proportion of simulations from each model accepted into the posterior probability distribution. If there are summary statistics included that add a lot of noise to the classification process, ABC will lose power in distinguishing support between models. As mentioned, RF is able to measure which summary statistics are the most influential in distinguishing between the models, through importance measures such as MDA and GINI. We used this information to select a subset of 10 summary statistics to be used in ABC model selection, along with a tolerance of 0.001 (Csilléry, François, & Blum, 2012). The performance of ABC in classifying the data can be measured using a cross‐validation approach for model selection which results in model misclassification rates for each model.

### 2.4 Power analyses

We compared the accuracy of three approaches in identifying community assembly models from the data simulated under the three community assembly models in CAMI. The first approach follows previous work and uses dispersion metrics, such as MPD and MNTD (standardized as NRI and NTI), in statistical hypothesis testing to infer the community assembly process from phylogenetic and phenotypic information, separately (Cornwell et al., 2006; Kembel et al., 2010; Kraft & Ackerly, 2010; Webb, 2000). For MNTD calculated using phenotypic information, the nearest neighbor is the species closest in trait space (Graham et al., 2012; Ricklefs & Travis, 1980; Swenson et al., 2012).

The second and third inference approaches are the approximate model selection techniques used in CAMI, RF (Breiman, 2001; Liaw & Wiener, 2002) and ABC (Csilléry et al., 2010, 2012; Toni, Welch, Strelkowa, Ipsen, & Stumpf, 2009). We measured the power of each approach in correctly classifying community assembly data (see Sections 2.1 and 2.2) through the OoB error rates for RF and model cross‐validation for ABC. We performed these power analyses for a range of community sizes to assess whether the power of any of the approaches increased with sample size of the regional/local community, which in this case is species richness. For data to classify, we simulated 1,000 datasets in CAMI under each community assembly model for 20 different regional community sample sizes ranging from 50 to 1,000, increasing by increments of 50, with the local community always half the size of the regional. For more details on each of the model identification techniques refer to Supplemental Methods Section 2.

We also investigated whether RF and ABC can be used to accurately infer the model of community and trait evolution simultaneously. For this, we performed the power analysis as described above, only here we classified six models (neutral, filtering, and competition models under both BM and OU models of trait evolution) rather than just the three community assembly models.

### 2.5 Parameter estimation

We measured the ability of the ABC approach to estimate the strength of the assembly process, $t_{E}\;\text{and}\;t_{C}$, under non‐neutral models of community assembly, environmental filtering, and competitive exclusion. For both models, we attempted parameter estimation when the traits were simulated under a BM and an OU model of trait evolution. We also attempted parameter estimation for two sizes of regional communities, 200 and 800, with corresponding local community sizes of 100 and 400. We simulated 50,000 community assembly datasets under each condition to serve as the reference dataset for parameter estimation. For details on these simulations refer the Supplemental Methods Section 3.

We simulated 100 datasets each for 13 different values of $t_{E}$ and $t_{C}$, ranging from 1 to 60 in increasing increments of 5 (see Supplemental Methods Section 3 for other parameter details). These simulated datasets would serve as the “observed” datasets to use for parameter estimation, in which case we know what the true value of $t_{E}$ and $t_{C}$ are. To measure not only how accurately $t_{E}$ and $t_{C}$ are estimated, but whether all values can be estimated accurately, we performed parameter estimation in ABC for each of the simulated datasets with a rejection algorithm and a tolerance of 0.001. For this, we assumed that data simulated under environmental filtering and competitive exclusion models were correctly classified as those models. We repeated this procedure increasing the sample size of the regional and local community to measure whether $t_{E}$ and $t_{C}$ estimates improved with increased sample size.

### 2.6 Empirical system

Craters of the Moon National Monument and Preserve (CRMO) is a volcanic landscape in southern Idaho. The overlapping basalt lava flows formed along vents in the Great Rift between 2 and 15 KYA (Kuntz, Champion, Spiker, & Lefebvre, 1986; Kuntz, Champion, Spiker, Lefebvrelsd, & Mcbroomes, 1982). Within the lava flows are kipukas—islands of vegetation that are completely surrounded by uninhabitable lava (Vandergast & Gillespie, 2004). Given their isolated nature and recent colonization, the plants on kipukas are an ideal system for studying community assembly. We opted to use maximum vegetative height as our functional trait of interest because it is known to be an important proxy for resource partitioning and competitive ability in plants (Cornwell et al., 2014; Weiher et al., 1999; Westoby, 1998).

The regional phylogeny was constructed for 113 species that occur in the CRMO by dropping non‐CRMO species (79,768) from a Spermatophyta phylogeny (Smith & Brown, 2017). Likewise, the local community phylogeny was constructed by dropping non‐kipuka community species from the regional phylogeny, resulting in 63 local species (Table S8). If a particular species needed was not in the Spermatophyta phylogeny, we used a random relative in the same genus as a replacement (Qian & Jin, 2016). In addition to the total local species pool on the kipukas, we also investigated eight kipukas individually, kipukas that consisted of 18–20 species from the local community (Table S10). Maximum vegetative height data for all species in the regional and local community were gathered using a combination of herbarium records, species descriptions, and floras (e.g., Hitchcock & Cronquist, 2018).

To assess whether an assembly process has structured the plant community on kipukas, we used NRI and NTI calculated from both phylogenetic and phenotypic (maximum vegetative height) information, separately, and CAMI using RF and ABC to perform model selection. We also performed parameter estimation using ABC to understand what the influence of $t_{E}$ or $t_{C}$ was on the assembly processes in either the filtering or competition models, should they be highly supported. For more details regarding the empirical data analysis, including plant collections and data simulated for the analysis, refer to the Supplemental Methods Sections 4.

## 3 RESULTS

### 3.1 Power analysis

**Figure 2:**
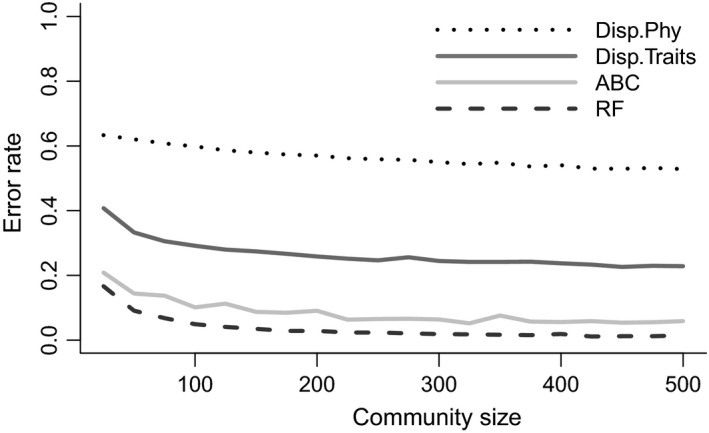
Error rates, or proportion of incorrectly classified simulations, when classifying community assembly models compared to the size of the local community used. Four model identification approaches are summarized here. The first is the average error rate when using dispersion metrics (MPD and MNTD) from phylogenetic information (dotted). The second is the average error rate when using dispersion metrics from functional trait information (black). The final two are model selection approaches employed in CAMI, ABC (gray), and RF (long dashed)

The average proportion of misclassified simulations using the standard approach of phylogenetic dispersion metrics for all regional/local community sizes was 56% (Table 1), decreasing from 63.3% to 52.9% with increasing sample size (Figure 2, Table S3). For each of the community assembly models, the average misclassification rate for each model was consistent between MPD and MNTD (Table 1) when using phylogenetic information. When calculating these metrics from phenotypic information, the average misclassification rate varied depending on whether MPD or MNTD was being used, with MPD having a very low error rate, 4.9%, and MNTD a high error rate, 48% (Table 1; Table S4).

**Table 1:** Average error rates for model classification approaches in classifying each of the three community assembly models, as well as overall classification error

	Neutral	Filtering	Competition	Mean
Phylogenetic				
MPD	4.810	72.590	90.845	56.082
MNTD	4.930	66.000	99.390	56.773
Phenotypic				
MPD	4.741	7.940	2.130	4.937
MNTD	4.911	39.855	99.465	48.077
RF	4.845	3.013	2.855	3.571
ABC	5.440	13.640	6.320	8.467

Average error rates for both model selection approaches were substantially lower. The average random forests OoB error rate when classifying community assembly models was 3.6%, ranging from 16.7% for small communities to 1.5% for large communities (Figure 2). The average OoB error rates for each community assembly model with RF were 4.8%, 3.0%, and 2.9% for neutral, filtering, and competition models, respectively (Table 1). The average ABC model misclassification rate was 8.47% (Table 1), ranging from 20.9% for small communities to 5.9% at large communities (Figure 2). The average ABC error rates for each community assembly model were 5.4%, 13.6%, and 6.32% for neutral, filtering, and competition models, respectively (Table 1).

Using RF and ABC to classify models of community assembly and trait evolution simultaneously resulted in overall higher error rates compared to inferring community assembly alone (Figure S1). On average, the average OoB error rate for RF was 23.2%, ranging between 45.7% and 16.2% from small to large communities (Table S5), and the overall error rate for ABC was 30.7%, ranging between 50.8% and 23.5% from small and large communities (Table S6).

### 3.2 Parameter estimation

**Figure 3:**
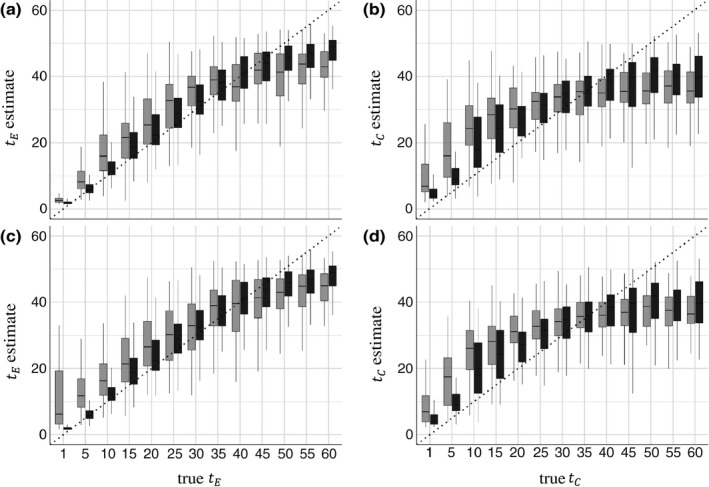
Estimation of $t_{E}$ and $t_{C}$ under their respective non‐neutral models of community assembly, coupled with one of two models of trait evolution. In each graph, the individual boxplots represent the median values of either $t_{E}\;\text{or}\;t_{C}$ from 100 independent attempts at parameter estimation, thus they are not posterior distributions, but rather a distribution of median parameter estimates. The *x*‐axis denotes the true value of $t_{E}\;\text{or}\;t_{C}$ simulated under. The light gray boxes represent datasets with regional/local community sizes of 200/100, and the dark gray boxes represent regional/local community sizes of 800/400. The dotted line in each plot represents a 1:1 correlation between estimated and true values of $\text{either}\;t_{E}\;\text{or}\;t_{C}$. (a) Environmental filtering community assembly with a BM model of trait evolution. (b) Competitive exclusion community assembly with a BM model of trait evolution. (c) Environmental filtering community assembly with an OU model of trait evolution. (d) Competitive exclusion community assembly with an OU model of trait evolution

For all models, the simulations with larger community sizes better estimated the true value of $t_{E}\;\text{and}\;t_{C}$ compared to communities of smaller size (Figure 3). Regardless of sample size, $t_{C}$ was overestimated when of smaller value. In both filtering and competition models, $t_{E}$ and $t_{C}$ are slightly underestimated when of larger value—though this is due to the true value of $t_{E}$ and $t_{C}$ being at the upper bound of the prior distribution, which if extended is less apparent.

### 3.3 Empirical system

Several dispersion metrics used from phylogenetic and phenotypic information identified significant under‐dispersion, or clustering, among plant species in the kipukas, suggesting a community assembly pattern of environmental filtering. When calculating NRI and NTI using phylogenetic information from all plants in the kipukas, the resulting *p*‐value was 0.02 for MPD and 0.29 for MNTD. When calculating the same metrics from phenotypes, the resulting *p*‐value for each test statistic was 0.03 and 0.01, respectively (Table S7). For the eight separate kipuka communities, only MPD using phylogenetic information identified two other communities as significantly under‐dispersed (Table S7).

We constructed two RF classifiers to make predictions about empirical data. One classifier was built with simulations from both trait models, and the other classifier was built with data simulated only under an OU trait model. This OU models‐only RF classifier was built because the trait data for the kipuka plants better fit an OU model of trait evolution compared to a BM model (see Supplemental Methods 4). The OoB error rates for these two classifiers were 25.50 and 23.61%, respectively. We also estimated the error rate when using ABC in the same way as with RF. For these, the error rate for each cross‐validation was 33.20 and 30.40%. Using these data and approaches, we predicted the model of community assembly for the empirical data with RF and ABC and saw a majority of support for environmental filtering, with the second highest support for the neutral model (Table 2 OU model‐only prediction, Table S11 for OU and BM model predictions).

**Table 2:** Community assembly model predictions from RF and model posterior probabilities from ABC for all local kipuka plant species and eight individual kipuka communities

	RF			ABC		
	Competition	Filtering	Neutral	Competition	Filtering	Neutral
ALL	–	0.64	0.36	–	0.82	0.18
B	0.06	0.54	0.4	–	0.35	0.65
C	0.06	0.6	0.34	–	0.5	0.5
D	0.07	0.61	0.32	–	0.92	0.08
E	0.06	0.58	0.36	–	0.67	0.33
F	0.02	0.46	0.52	–	0.47	0.53
G	0.05	0.52	0.43	–	0.6	0.4
H	0.04	0.52	0.44	0.02	0.47	0.52
I	0.08	0.48	0.45	0.32	0.25	0.43

Note

All predictions were made with simulations using an OU model of trait evolution.

**Figure 4:**
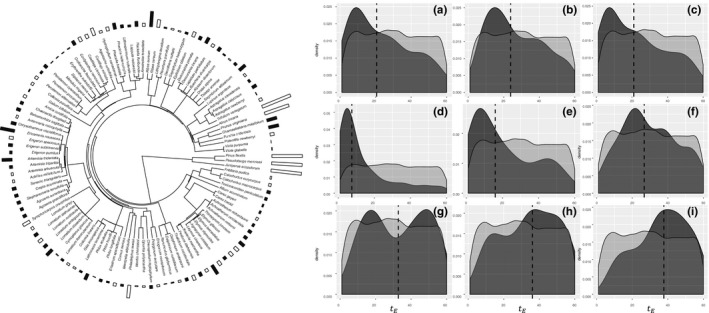
(left) Regional phylogeny of species in the Craters of the Moon National Monument and Preserve, coupled with each species' maximum vegetative height in meters represented by the filled bar plots by each species. Species only present in the regional community have their trait bars colored white, while species that are also present in the local community have their trait bars colored black. The bars are truncated at 6 m, as only the four trees in this study are larger than 6 m, and those species and their heights are available in Table S8. (right) Nine panels displaying the prior (light gray) and posterior (dark gray) probability distributions of $t_{E}$ under an environmental filtering model and OU model trait evolution. The dotted line represents the median estimate of $t_{E}$. (a) Estimate from the entire local kipuka plant species pool. (b–i) Estimates from the separate eight kipuka communities

We performed parameter estimation of $t_{E}$ for the environmental filtering model for each dataset under an OU model of trait evolution (Table S12). Each time 100 simulations were accepted as from the posterior distribution of $t_{E}$ (Figure 4). We also compared the amount of model support for the environmental filtering models with the median estimate of $t_{E}$ (Figure S2, Table S12) to show the relationship between the strength of the filtering process and the model support received.

## 4 DISCUSSION

### 4.1 Performance of CAMI

Using CAMI, we can correctly classify models of community assembly and, importantly, quantify the uncertainty associated with community assembly model inference. This approach improves upon current methods in community phylogenetics by harnessing the critical information present in the phenotypic and phylogenetic data that directly relate observed patterns to processes. Our approach is successful, in part, because over‐ and under‐dispersion in the phylogenetic and trait data are emergent properties of the community assembly models described. Through our method, we can control the processes that directly impact the amount of over‐ and under‐dispersion in the phenotypic data, along with their degree of association with the phylogenetic information. Furthermore, our inference pipeline is unique in allowing users to gauge or rank evidence for both neutral and non‐neutral assembly processes.

The performance of RF and ABC is comparable in that they both accurately classify the community assembly models. A benefit to using RF is that all of the summary statistics from the simulated data can be used without compromising the power or computational speed of the method. Additionally, RF measures how important each summary statistic is for classifying the data accurately. While we do not use this information for any additional community assembly inferences here, there is potential to ask which summary statistics play an important role in these assembly processes, and further, whether there are any biological implications to gain from that information. The main advantage of using ABC is that parameter estimation is straight forward using simulated data, and this is particularly relevant for estimating the strength of non‐neutral assembly via $t_{E}$ and $t_{C}$, though parameter estimation using RF is increasingly common.

The predictive approaches outlined here are not meant to replace dispersion metrics, but rather to be used as an additional tool in making inferences about community assembly. We have shown here, as others have (Kraft et al., 2007), that dispersion metrics are not reliable in determining models of community assembly with phylogenetic information alone. When using phenotypic data though, MPD proved to be comparable in accuracy at distinguishing community assembly models to RF and ABC, though MNTD still had very high error rates (Table 1).

Though CAMI is currently implemented using one trait, the analyses do not necessarily need to be limited to one trait. If there are several traits of interest in a particular community, data dimension reduction techniques could be used, such as principle components or linear discriminate analysis, to associate each species with a singular value representing where they fall in trait space with respect to other species in the community. Though we do not explore the power of inferring models of community assembly from several traits defined in one composite dimension through simulations, we expect, to some degree, that the method will behave as presented above in the single‐trait case. Using multiple traits in a true multivariate framework, which we have not implemented, could make for an even more powerful inference, as many factors influencing community structure could be measured at once (Herben & Goldberg, 2014; Kraft et al., 2015; Weiher, Clarke, & Keddy, 1998). However, if multiple traits are being considered, there also need be the consideration that there could be multiple phenotypic optima or complex routes of competition between species, and here, we consider the presence of only a single optimum and equal competition among species (Weiher et al., 1998, Marks & Lechowicz, 2017).

While we feel CAMI will continue to make progress in advancing our understand of community ecological patterns globally, there are still many aspects of community ecological theory yet to be incorporated (Belyea & Lancaster, 1999; Weiher et al., 2011). The assembly models defined here could be made more powerful by considering other community dynamics such speciation, colonization, and extinction during the assembly process (Rosindell & Harmon, 2013), as well as co‐occurring and structured non‐neutral processes (Keddy & Shipley, 1989) where the relative importance of these processes can be measured (as in Munoz et al., 2018; van der Plas et al., 2015). These aspects may be more or less relevant depending on the taxonomic scale of the community being investigated (Weiher et al., 2011). Furthermore, the inference power could expand by making CAMI an individual‐based model of community assembly (Pontarp et al., 2019; Rosindell, Harmon, & Etienne, 2015), where individuals can diverge to speciate and harbor intraspecific diversity among phenotypes (Jung et al., 2014; Jung, Violle, Mondy, Hoffmann, & Muller, 2010), all while abundance distributions and population demographics are being tracked (HilleRisLambers et al., 2012; Overcast, Emerson, & Hickerson, 2019). A spatially explicit model (see Pontarp et al., 2019) could allow for the exploration of how geography, or even local topography, impacts the assembly process. Ultimately, we believe this approach has the capability of being extended to incorporate many more complexities known to influence and emerge from the assembly process.

### 4.2 Inferring the strength of the assembly process

Parameterizing the strength of the assembly process provides an additional mode of inference for the relative strength of the non‐neutral community assembly processes, environmental filtering, $t_{E}$, and competitive exclusion, $t_{C}$. We have shown that ABC can be an appropriate tool to estimate both $t_{E}$ and $t_{C}$ accurately (Figure 3) for their respective community assembly models. We have also shown that empirical data, from different communities, do indeed bear some signal to indicate different magnitudes of $t_{E}$ (Figure 4). Additionally, we show that the estimate of $t_{E}$ has a relationship with the amount of support the corresponding non‐neutral model receives, in this case, the environmental filtering model. We know that for filtering models, the smaller the value of $t_{E}$, the stronger the effects of filtering, thus the smaller the estimate of $t_{E}$, the greater the model support for environmental filtering (Figure S2). Having this measure that can quantify the influence of the assembly process at play opens the door for comparisons of communities globally that have been assembled by the same mechanism (Götzenberger et al., 2012). Prior to now, if multiple communities were inferred to be assembled via environmental filtering, there was no way to ask whether one environment's pressure was stronger relative to the other, while $t_{E}$ and $t_{C}$ now permits these questions.

### 4.3 Models of trait evolution

Identifying models of community assembly alone were much more successful than when trying to simultaneously identify models of trait evolution, as shown by the increase in error rates (Figure S1). When the model of trait evolution is identifiable, as in many BM and OU cases, simulating under both models is not necessary as it drastically increases the amount of simulations needed. Information about the best fit trait model, including parameter estimates, can be used to directly inform parameters used to simulate community assembly data in CAMI (as in the empirical study here). However, we do show that considering both models of trait evolution simultaneously versus only one at a time does not drastically change the community assembly inference (Table S11). Thus, should one be unable to properly, or with confidence, estimate the true model of trait evolution, the combined inference procedure in CAMI is appropriate, and this may be especially useful for early‐burst or multi‐optima OU models of trait evolution (Slater & Pennell, 2013; Uyeda & Harmon, 2014). We should note here that a model of trait evolution fit to community data, phylogenetic and phenotypic, involves excluding many taxa from the tree and trait distributions that would otherwise be included in phylogenetic comparative methods. This means the parameter estimates cannot be tied to the entire evolution of a particular trait, but rather its evolution among a certain set of species within a community.

### 4.4 Empirical inference

When using CAMI to distinguish models of community assembly, a majority of support reliably goes to the environmental filtering model when considering the entire local kipuka community, with some support garnered by the neutral model (Table 2). When looking at the eight separate kipuka communities, the environmental filtering model still receives a majority of the support, but there is quite a lot of support for the neutral model as well, and sometimes even for the competitive exclusion model (Table 2). Conveniently though, when comparing the model probability estimates with the $t_{E}$ estimates, we get a better understanding of why the model support is where it is for a particular kipuka and that the $t_{E}$ parameter is being estimated appropriately (Figure S2). Essentially, when $t_{E}$ is representing weaker filtering effects, which corresponds to higher values of $t_{E}$, we see lower support for the filtering models.

When using dispersion metrics to distinguish models of community assembly, the reliability is less apparent. Many of the observed dispersion metrics fall at the lower ends of the random distribution of dispersion indices and subsequently result in low *p*‐values. However, one of the caveats of hypothesis testing is that there is an arbitrary cutoff between when something is significant and when it is not that is predetermined by the user. In this case, technically the cutoff is .025 and so only four out of 36 metrics were significant. These issues are generally overcome with intuition because it is obvious some of the *p*‐values are still very low, but they do highlight problems with hypothesis testing and relying on *p*‐values for marks of biological significance.

For each kipuka species pool, the strength of the filtering process was estimated quite differently. For the entire species pool of the kipukas, the $t_{E}$ estimate was a relatively moderate value, 15.4, given the prior range of 1–60, where values near 1 imply strong filtering, and values closer to 60 imply weak filtering. For other kipuka communities though, $t_{E}$ was often a moderate estimate, falling somewhere in the middle of the prior distribution, though sometimes the estimate was very low (Figure 4d,e) and other times, quite high (Figure 4i). We recognize though that any interpretation of $t_{E}$ is challenging because the parameter has never before been measured using any community or trait before. Thus, we expect with continued investigations of community data using CAMI we will decipher a sharper picture on how $t_{E}$ behaves across many natural communities. These estimates are a start to that investigation given their correspondence with the model probabilities (Figure S2). We should note that in the case of these $t_{E}$ estimates, the rate of character change is so low that a strong effect of filtering with that little phenotypic variation may be harder to detect than if more variation were present. Similarly, the estimates of $t_{E}$ are less reliable when the community size is small (Figure 3), which is true in the case of these kipukas.

One anecdotal explanation for the support for the environmental filtering assembly model lies in the structure of the kipukas. Lava flow builds up on the edges of the habitable land on the kipuka forming a sort of “bowl,” with the plant community inside the bowl. Species that generally grow taller than the bowl edges are less protected from heavy wind speeds common in the area and are more likely to be filtered from the environment. Likewise, with high wind speed comes a likely increase in dispersal ability for some species in the regional pool, which may explain the support of the neutral model. However, even though we can speculate on the cause for the support of an environmental filtering model acting on height in the kipukas, we still lack evidence of the true cause of the support, or mechanism of filtering.

While vegetative height has been hypothesized to play an important role in community structure, as a functional phenotype and a proxy for other important traits (Cornwell et al., 2014), because we only take into account a single functional trait, we recognize the potential limitations to these inferences. The CAMI framework permits testing multiple traits independently and comparing the evidence across how each trait influenced community assembly to better understand the historical and contemporary assembly processes (Herben & Goldberg, 2014). Additionally, each trait, if influencing community assembly in a non‐neutral way, will be associated with an estimate of $t_{E}$ or $t_{C}$, which will also provide insight into the degree that each trait influences the assembly process for a particular community.

## 5 CONCLUSION

CAMI is a new approach able to estimate the probability of neutral and non‐neutral community assembly models given observed phylogenetic and phenotypic information. By harnessing the power of simulations and approximate approaches for model selection, such as RF and ABC, we can quantify uncertainty in community assembly inferences. Additionally, new parameters described here, $t_{E}$ and $t_{C}$, govern the strength of environmental filtering and competition models, respectively, and are estimable with empirical data. Defining the non‐neutral assembly models and parameterizing the processes to mimic strong to mild assembly dynamics will add to what we know about communities that have been assembled via the same mechanisms. While there are other approaches that infer community assembly in a model‐based framework (Munoz et al., 2018; Pontarp et al., 2019; van der Plas et al., 2015), CAMI offers a unique opportunity to use information that is readily available in phylogenetic community ecology. Given these data are common for community assembly studies, this framework could be readily applied to many existing systems and ultimately provides information about the patterns of community assembly globally.

## CONFLICT OF INTEREST

None declared.

## AUTHOR CONTRIBUTIONS

MR, DCT, and LJH developed research concept. BW contributed to the creation of the non‐neutral assembly models and KP collected all empirical data. MR developed CAMI, performed all analyses, and wrote the manuscript. All authors contributed to critiques of the analysis and subsequent revisions of the text.

### Open Research Badges


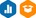
This article has been awarded https://openscience.com and https://openscience.com Badges. All materials and data are publicly accessible via the Open Science Framework at https://github.com/ruffleymr/CAMI/tree/master/data and https://github.com/ruffleymr/CAMI.

## Data Availability

All code for the R package CAMI is available at https://github.com/ruffleymr/CAMI. All scripts for each analysis, along with the output data, can be found in https://github.com/ruffleymr/CAMI/vignettes and https://github.com/ruffleymr/CAMI/data, respectively.
