# Supplementary material for: Identifying models of trait‐mediated community assembly using random forests and approximate Bayesian computation
Source: Ecol Evol. 2019 Nov 21;9(23):13218–30. doi: 10.1002/ece3.5773 (PMC6912896; doi:10.1002/ece3.5773)
Supplement: Supplementary file 1 [file ECE3-9-13218-s001.docx]

**Supplemental Material**

**Supplemental Methods**

1. *Prior Ranges for Data Simulation*

For the data used in each model classification approach, for the power analyses, we simulated 1,000 datasets under each community assembly model and trait evolution model (6 models), for 20 different regional community sample sizes ranging from 50 to 1000, increasing by increments of 50, for a total of 120,000 simulations. In each of these simulations, the local community size, $n$, was exactly half the size of the regional community, $N$. The speciation, $\lambda$, extinction fraction (equal to $\mu/\lambda$), rate of character change, $\sigma^{2}$, and, for OU models, strength of phenotypic pull, $\alpha,$ parameters were all drawn from a default prior distribution that was consistent for all simulations (Supplemental Table 1). Default priors were selected because we believed they were reasonable representations of where community phylogenetic and functional trait data exist currently. Simulations of non-neutral models included the strength of assembly, $t$, parameter that is drawn from a uniform prior distribution between 1 and 60. This distribution roughly bounds the probability of persistence, $P_{z_{i},z_{E}}$ for environmental filtering and $P_{z_{i},\bar{z}}$ for competitive exclusion, at a minimum of 0.001 and a maximum of 0.99. These minimum and maximum bounds will vary slightly for each simulation depending on $\sigma^{2}$ and, in the case of OU models, $\alpha$.

1. *Power Analyses: model identification techniques*

*2.2 Dispersion Metrics* – For the first model identification technique, NRI and NTI (Webb *et al.* 2002, 2008; Kembel *et al.* 2010) were used to measure phylogenetic dispersion. Null distributions for each test statistic were generated by shuffling the taxon labels of the phylogenetic distance matrix (Webb *et al.* 2008; Kembel *et al.* 2010). We assumed the traits were phylogenetically conserved, thus if either test statistic were significantly greater than expected, *i.e.* p-value $\geq$ 0.975 and phylogenetic over-dispersion, competitive exclusion was inferred. If either test statistic were significantly lower than expected, *i.e.* p-value $\leq$ 0.025 and phylogenetic under-dispersion, environmental filtering was inferred. If both test statistics were non-significant, a neutral community assembly model was inferred. We totaled the number of correctly classified simulations to calculate the proportion of misclassified simulations and report the overall error rate of the approach.

We also calculated the standard effect size of MPD and MNND (in the same way NRI and NTI are calculated) to measure phenotypic dispersion. For this, instead of using phylogenetic distances, distances in phenotypic space were used to calculate MPD and MNND, along with the null distributions (Cornwell *et al.* 2006; Kraft & Ackerly 2010). We made the same assumptions about the traits being phylogenetically conserved and therefore followed the same inference procedure as with phylogenetic information.

*2.3 Random Forests* – When performing model selecting using RF, all 30 summary statistics (Supplemental Table 2) we used in constructing a ‘forest’ of 1000 decision trees. Each tree classifies the simulated data as from either a neutral, environmental filtering, or competitive exclusion model of community assembly, essentially each tree casts a “vote” for a model. The proportion of votes a model receives is considered the probability of that model, meaning the model with the most votes, or highest probability, is the model selected. Each decision tree is constructed using the summary statistics from a random 2/3 of the data, while the remaining 1/3 of data, or out-of-bag (OOB) data, is used to cross-validate the accuracy of each decision tree. Through this, the error rate of each model is estimated and summarized in the OOB error rates. These error rates indicate the overall accuracy of the forest in distinguishing between the models.

*2.4 Approximate Bayesian Computation* – To determine the overall model misclassification rate when using ABC for model selection, we performed 500 cross-validation attempts for each community assembly model for each of the 20 regional community sample sizes. For each cross-validation, the rejection algorithm was used with a tolerance of 0.01. The total proportion of simulations that were incorrectly classified for each model were averaged as an overall error rate for each regional community sample size. Mean posterior probabilities were calculated amongst the correctly classified models as an additional measure of how much support the correct model received.

1. *Reference Data Simulation for Parameter Estimation*

We simulated 50,000 community assembly datasets under each condition to serve as the reference dataset for parameter estimation. In these simulations, the regional and local community sizes were fixed using the values stated above. A uniform prior was set on $\sigma^{2}$ to be between 2 and 4, which is narrower than the default uniform prior between 1 and 10. This was done because if one has the empirical phenotypic data, they are typically able to estimate a rate of trait evolution under BM or OU models of trait evolution, and would thus have more information for this parameter and not need to rely on the default uniform distribution. The remaining parameters $t, \lambda, \mu,$ and $\alpha$ were drawn from their default uniform prior distributions.

1. *Empirical System*

Voucher plant specimens were collected during the summer of 2016 and 2017 across 25 kipukas throughout the CRMO. In total, 63 unique plant species were documented as occurring in these CRMO kipukas, and these species represented the total local plant community. To assemble the regional community species list, we referenced a checklist of the vascular plants that occur in the CRMO (Popovich 2006). We selected an additional 50 species on the basis of their abundance and whether they had phylogenetic representation (see below) from this checklist to be included as species in the regional community. In addition to analyzing the total local species pool as a community, we also investigated eight kipuka communities separately that had a subset of 18-20 species from the local species pool.

To assess whether an assembly process has structured the plant community on kipukas, we used the standardized effect size of MPD and MNTD using phylogenetic information, MPD and MNND using phenotypic (maximum vegetative height) information, and CAMI using RF and ABC to make model predictions. We also performed parameter estimation using ABC to understand what the influence of $t_{E}$or $t_{C}$ was on the assembly processes in either the filtering or competition models that were supported. Again, this was done on the total local species pool in the kipukas, as well as the separate eight kipuka plant communities (Supplemental Table 10).

When using trait information, the maximum vegetative plant heights were log transformed because the data were strongly right skewed, which did not coincide with the simulated data. For analysis using dispersion metrics, we performed 1000 iterations of a random community to include in the null distribution and each random community was constructed by shuffling the taxon labels of either the phylogenetic or phenotypic distance.

Before simulating data for RF and ABC model selection, we determined the best fit model of trait evolution for the empirical data, given we have the regional phylogeny and all phenotypic (maximum vegetative height) information for each individual. This information was used to constrain the model of trait evolution and $\sigma^{2}$, and potentially $\alpha$, that we simulated community assembly data under. The OU model of trait evolution was always selected over a BM model (Supplemental Table 9), though the estimation of precise values for $\sigma^{2}$and $\alpha$ proved challenging because we could only estimate the quotient of $\sigma^{2}$and $\alpha$ (Hansen 1997; Butler & King 2004) (Supplemental Table 9).

For analysis using CAMI, we simulated 60,000 community assembly datasets under all models of community assembly and trait evolution, 10,000 per model, to use for model selection. The number of regional and local species in each simulation was fixed to 113 and 63, respectively, to mimic the empirical data. We opted to use the combination of $\sigma^{2}$and $\alpha$, where $\alpha$ was a reasonably estimate at 0.02 and $\sigma^{2}, 0.77$; both parameters were fixed to these values. The other parameters $t, \lambda, and \mu$were drawn from their default uniform prior distributions (Supplemental Table 1).

These data and the empirical data, from the total kipuka community and the eight separate kipukas, were summarized into 30 summary statistics (Supplemental Table 2) to be used for RF and ABC. For RF, we constructed a classification forest, or classifier, of 5000 decision trees using all 60,000 simulations and 30 summary statistics. We then used this classifier to predict which model of community assembly structured the kipuka plant community through vegetative height. For ABC, we used the top 10 summary statistics from the RF classifier (as in sections *3.4* and *3.5*) and all 60,000 simulations to estimate the posterior probability of each model given the data. For both RF and ABC, we predicted the model probabilities while only considering the community assembly models (neutral, filtering, competition), but then also while the model of trait evolution (BM and OU).

We performed parameter estimation using ABC to understand what the influence of $t_{E}$or $t_{C}$ was on the assembly processes in either the filtering or competition models that were supported for each kipuka dataset. For the supported models, we simulated 50,000 community assembly datasets under the default uniform priors for parameters $t, \lambda, \mu,$ and $\alpha$, with a narrower prior distribution on $\sigma^{2},$ centering the empirically estimated $\sigma^{2}$. We always accepted 100 simulations as from the posterior for parameter estimation.

**Supplemental Results**

*1.0 Importance of Summary Statistics*

The summary statistics that RF determined to be most informative were the difference in variance of trait values between the local and regional communities, the variance of the local traits, the kurtosis of the local traits, the variance of the regional traits, the bimodal coefficient calculated from the local traits, the difference in the mean of the trait values between the local and regional community, the difference between the normalized Lineage-Through-Time statistic (Janzen *et al.* 2015) calculated for both the local community phylogeny and regional community phylogeny, the slope of a linear model fitted to the absolute value of the phylogenetic independent contrasts against their expected variances (following Garland *et al.* 1992), mode length from the local trait distribution, and finally, the slope of a linear model fitted to the absolute value of the contrasts against node depth (after Purvis & Rambaut 1995).

**Supplemental Figures**


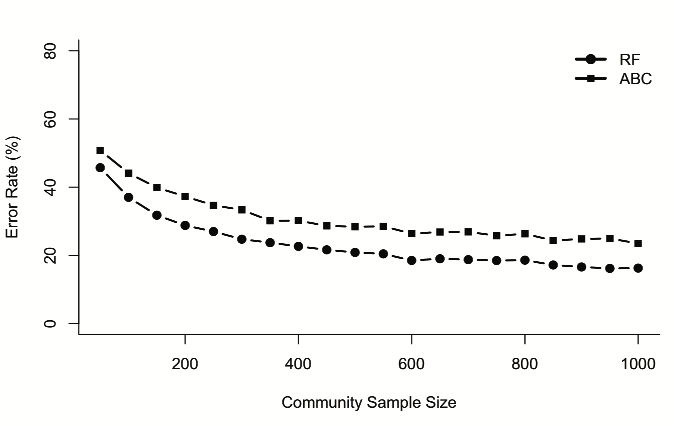


**Supplemental Figure 1.** Error rates, or proportion of incorrectly classified simulations, when classifying community assembly and trait evolution models compared to the size of the local community used. Square symbols indicate RF was used to classify data and circle symbols indicate ABC was used to classify data.

**Supplemental Figure 2.** Linear regression models between the model support for environmental filtering, as predicted from RF and ABC) and the $t_{E} median estimates from ABC$. The correlation coefficient when comparing RF model support values and $t_{E} estimates was 0.65$, and when comparing with ABC the coefficient was 0.89.

**Supplemental Tables**

**Supplemental Table 1.** Default parameter prior distributions used for simulating data in CAMI.

**Supplemental Table 2.** Summary statistics calculated in CAMI and used for model selection in RF.

**Supplemental Table 3.** The average proportion of misclassified simulations using the standard approach of phylogenetic dispersion metrics for all regional/local community sizes tested and for each model of community assembly.

**Supplemental Table 4.** The average proportion of misclassified simulations using phenotypic dispersion metrics for all regional/local community sizes tested and for each model of community assembly.

**Supplemental Table 5.** Average error rates, or proportion of incorrectly classified simulations, when classifying community assembly and trait evolution models using random forest for all sizes of the local community used.

**Supplemental Table 6.** Average error rates, or proportion of incorrectly classified simulations, when classifying community assembly and trait evolution models using ABC for all sizes of the local community used.

**Supplemental Table 7.** Results for empirical data when using dispersion metrics MPD and MNTD with phylogenetic and phenotypic information for the total kipuka plant community and the eight individual kipuka plant communities. The mean and standard deviation of the null distribution are included, as well as the p-value for the observed value’s position in the null distribution. Significant p-values are indicated with **.

**Supplemental Table 8.** All species included in the regional and local community phylogeny for the kipukas od CRMO. Species that were present in the community but not in the Spermatophyta phylogeny were replaced in the phylogeny with a close relative, denoted in the table. The minimum, maximum, and range of vegetative height for each species is also denoted in cm.

| **Genera** | **specific epithet** | **Species from Smith Tree (if replacement is needed)** | **species min height (cm)** | **species max height (cm)** | **species range height (cm)** |
| --- | --- | --- | --- | --- | --- |
| Achnatherum | lemmonii | Achnatherum richardsonii | 20 | 70 | 20-70 |
| Achnatherum | thurberianum | Achnaterum nelsonii | 30.5 | 70 | 30.5-70 |
| Acnatherum | occidentale |  | 25 | 45 | 25-45 |
| Agoseris | aurantiaca |  | 10 | 60 | 10-60 |
| Agoseris | glauca | Agoseris grandiflora | 10 | 70 | 10-70 |
| Allium | acuminatum |  | 10 | 30 | 10-30 |
| Alyssum | desertorum |  | 10 | 25 | 10-25 |
| Artemisia | tridentata |  | 40 | 200 | 40-200 |
| Artemisia | tripartita |  | 20 | 60 | 20-60 |
| Astragalus | filipes | Astragalus calycosus | 30 | 90 | 30-90 |
| Astragalus | lentiginosus | Astragalus canadensis | 10 | 40 | 10-40 |
| Astragalus | purshii | Astragalus newberryi | 5 | 10 | 5-10 |
| Balsamorhiza | sagittata |  | 15 | 80 | 15-80 |
| Boechera | divaricarpa |  | 25 | 80 | 25-80 |
| Calochortus | macrocarpus |  | 20 | 70 | 20-70 |
| Carex | filifolia | Carex geyeri | 5 | 35 | 5-35 |
| Chaenactis | douglasii |  | 10 | 60 | 10-60 |
| Chamaebatiaria | millefolium |  | 100 | 200 | 100-200 |
| Chenopodium | leptophyllum |  | 20 | 60 | 20-60 |
| Chrysothamnus | viscidiflorus |  | 20 | 100 | 20-100 |
| Cordylanthus | ramosus |  | 10 | 90 | 10-90 |
| Crepis | acuminata |  | 20 | 70 | 20-70 |
| Delphinium | andersonii |  | 30 | 60 | 30-60 |
| Descurainia | incana |  | 15 | 120 | 15-120 |
| Descurainia | pinnata |  | 10 | 70 | 10-70 |
| Diplacus | nanus |  | 1.3 | 10 | 1.3-10 |
| Elymus | elymoides |  | 10 | 60 | 10-60 |
| Eriastrum | sparsiflorum |  | 5 | 30.5 | 5-30.5 |
| Ericameria | nauseosa |  | 20 | 280 | 20-280 |
| Erigeron | pumilus |  | 5 | 50 | 5-50 |
| Erigeron | subtrinervis |  | 15 | 80 | 15-80 |
| Eriogonum | caespitosum |  | 3 | 10 | 3-10 |
| Erythranthe | suksdorfii |  | 3 | 10 | 3-10 |
| Festuca | viridula | Festuca idahoensis | 40 | 80 | 40-80 |
| Galium | bifolium |  | 5 | 20 | 5-20 |
| Gayophytum | ramosissimum | Gayophytum heterozygum | 15 | 40 | 15-40 |
| Hackelia | floribunda |  | 30 | 100 | 30-100 |
| Lappula | redowskii |  | 5 | 40 | 5-40 |
| Lathrocasis | tenerrima |  | 3 | 35 | 3-35 |
| Leymus | cinereus |  | 100 | 200 | 100-200 |
| Lithophragma | tenellum |  | 10 | 25 | 10-25 |
| Lithospermum | ruderale |  | 20 | 60 | 20-60 |
| Lomatium | ambiguum |  | 10 | 80 | 10-80 |
| Lomatium | foeniculaceum |  | 3 | 40 | 3-40 |
| Lomatium | multifidum | Lomatium dissectum | 50 | 150 | 50-150 |
| Lomatium | simplex | Lomatium idahoense | 20 | 80 | 20-80 |
| Mentzelia | albicaulis |  | 10 | 40 | 10-40 |
| Penstemon | cyananthus |  | 30 | 70 | 30-70 |
| Penstemon | procerus |  | 5 | 40 | 5-40 |
| Phacelia | heterophylla |  | 20 | 120 | 20-120 |
| Phlox | aculeata |  | 61 | 122 | 61-122 |
| Phlox | hoodii |  | 3 | 6 | 3-6 |
| Phlox | longifolia |  | 10 | 50 | 10-50 |
| Purshia | tridentata |  | 100 | 200 | 100-200 |
| Ranunculus | glaberrimus |  | 5 | 20 | 5-20 |
| Ribes | aureum |  | 100 | 300 | 100-300 |
| Senecio | sphaerocephalus | Senecio trinagularis | 30 | 80 | 30-80 |
| Sisymbrium | altissimum |  | 30 | 150 | 30-150 |
| Stephanomeria | tenuifolia |  | 20 | 70 | 20-70 |
| Thinopyrum | intermedium | Thinopyrum junceum | 91.5 | 122 | 91.5-122 |
| Thlaspi | arvense |  | 10 | 50 | 10-50 |
| Toxicoscordion | paniculatum |  | 30 | 50 | 30-50 |
| Viola | nuttallii | Viola glabella | 3 | 12 | 3-12 |
|  |  |  |  |  |  |
| Achillea | millefolium |  | 20 | 40.5 | 20-40.5 |
| Agastache | urticifolia |  | 40 | 150 | 40-150 |
| Agropyron | cristatum |  | 30.5 | 91.5 | 30.5-91.5 |
| Alnus | incana |  | 460 | 2500 | 460-2500 |
| Amsinckia | tessellata |  | 15 | 60 | 15-60 |
| Antennaria | microphylla |  | 5 | 40 | 5-40 |
| Arnica | cordifolia |  | 10 | 60 | 10-60 |
| Artemisia | arbuscula |  | 10 | 40 | 10-40 |
| Calochortus | eurycarpus |  | 10 | 50 | 10-50 |
| Carex | douglasii | Carex backii | 15 | 46 | 15-46 |
| Castilleja | chromosa |  | 30.5 | 90 | 30.5-90 |
| Castilleja | miniata |  | 30.5 | 80 | 30.5-80 |
| Chrysothamnus | viscidiflorus |  | 20 | 100 | 20-100 |
| Collinsia | parviflora |  | 5 | 50 | 5-50 |
| Collomia | linearis |  | 10 | 40.5 | 10-40.5 |
| Cornus | sericea |  | 20 | 60 | 20-60 |
| Cymopterus | glaucus |  | 2 | 15 | 2-15 |
| Delphinium | nuttallianum |  | 15 | 40 | 15-40 |
| Draba | verna | Draba alpina | 5 | 20 | 5-20 |
| Elymus | glaucus |  | 50 | 100 | 50-100 |
| Elymus | lanceolatus |  | 30.5 | 91.5 | 30.5-91.5 |
| Epilobium | ciliatum |  | 30 | 100 | 30-100 |
| Erigeron | speciosus |  | 15 | 80 | 15-80 |
| Fritillaria | pudica |  | 10 | 30 | 10-30 |
| Galium | bifolium |  | 5 | 20 | 5-20 |
| Gilia | inconspicua |  | 8 | 32 | 8-32 |
| Heuchera | parvifolia |  | 23 | 30.5 | 23-30.5 |
| Hydrophyllum | capitatum | Hydrophyllum canadense | 10 | 46 | 10-46 |
| Juniperus | scopulorum |  | 100 | 1000 | 100-1000 |
| Koeleria | macrantha | Koeleria macrantha | 30 | 60 | 30-60 |
| Lepidium | perfoliatum |  | 20 | 60 | 20-60 |
| Lomatium | grayi |  | 15 | 50 | 15-50 |
| Lomatium | nudicaule |  | 20 | 90 | 20-90 |
| Lupinus | argenteus |  | 10 | 40.5 | 10-40.5 |
| Mimulus | nanus | Mimulus ringens | 1.25 | 10 | 1.25-10 |
| Montia | chamissoi |  | 5 | 20 | 5-20 |
| Oenothera | caespitosa | Oenothera nuttallii | 7.5 | 23 | 7.5-23 |
| Opuntia | polyacantha |  | 10 | 30.5 | 10-30.5 |
| Orobanche | fasciculata |  | 3 | 15 | 3-15 |
| Penstemon | deustus | Penstemon attenuatus | 20 | 60 | 20-60 |
| Phacelia | hastata |  | 25.5 | 76 | 25.5-76 |
| Philadelphus | lewisii |  | 150 | 250 | 150-250 |
| Pinus | flexilis |  | 396 | 1524 | 396-1524 |
| Polygonum | douglasii | Polygonum aviculare | 10 | 40 | 10-40 |
| Potentilla | gracilis | Potentilla newberryi | 40 | 80 | 40-80 |
| Prunus | virginiana |  | 100 | 500 | 100-500 |
| Pseudotsuga | menziesii |  | 2133.5 | 9144 | 2133.5-9144 |
| Ribes | cereum |  | 50 | 150 | 50-150 |
| Symphoricarpos | oreophilus |  | 50 | 150 | 50-150 |
| Thalictrum | occidentale |  | 40 | 100 | 40-100 |
| Trifolium | variegatum |  | 10 | 60 | 10-60 |
| Viola | purpurea |  | 0.5 | 1.5 | .5-1.5 |

**Supplemental Table 9.** Parameter estimates, log-likelihoods and AIC scores for BM and OU models of trait evolution. The BM model of trait evolution was optimized one time, with the resulting log-likelihood and AIC. For the OU model, we struggled to estimate the parameter values absolutely. However, we could estimate the quotient of $\sigma^{2}$and $\alpha$ and did so for 21 parameter combinations. Regardless of the $\sigma^{2}$and $\alpha$ though, OU was always a better fit model.

**Supplemental Table 10.** Parameter Presence/Absence matrix indicating which kipuka species is present on one of the eight specific kipukas investigated. The table contains all of the species that occur in the local kipuka community.

| **Species (Smith Tree Replacements Included)** | **Kipuka 16** | **Kipuka 11** | **Kipuka 671** | **Kipuka 425** | **Kipuka 3** | **Kipuka 620** | **Kipuka Pratt1** | **Kipuka 18** |
| --- | --- | --- | --- | --- | --- | --- | --- | --- |
| **Figure Reference** | **B** | **C** | **D** | **E** | **F** | **H** | **I** | **J** |
| *Achnatherum_richardsonii* | 1 | 1 | 0 | 0 | 0 | 0 | 0 | 0 |
| *Achnatherum_nelsonii* | 0 | 0 | 1 | 1 | 0 | 0 | 0 | 0 |
| *Achnatherum_occidentale* | 0 | 0 | 0 | 0 | 0 | 0 | 0 | 0 |
| *Agoseris_aurantiaca* | 0 | 0 | 0 | 0 | 0 | 0 | 0 | 0 |
| *Agoseris_grandiflora* | 1 | 1 | 1 | 1 | 0 | 0 | 0 | 0 |
| *Allium_acuminatum* | 0 | 0 | 1 | 1 | 1 | 0 | 0 | 0 |
| *Alyssum_desertorum* | 0 | 0 | 0 | 0 | 0 | 1 | 0 | 0 |
| *Artemisia_tridentata* | 1 | 0 | 1 | 1 | 1 | 1 | 1 | 1 |
| *Artemisia_tripartita* | 1 | 1 | 0 | 0 | 0 | 0 | 1 | 1 |
| *Astragalus_calycosus* | 1 | 1 | 0 | 0 | 0 | 0 | 0 | 0 |
| *Astragalus_canadensis* | 0 | 0 | 0 | 1 | 0 | 0 | 0 | 0 |
| *Astragalus_newberryi* | 0 | 0 | 0 | 0 | 0 | 1 | 1 | 0 |
| *Balsamorhiza_sagittata* | 1 | 1 | 0 | 1 | 1 | 0 | 0 | 0 |
| *Boechera_divaricarpa* | 1 | 0 | 0 | 0 | 1 | 1 | 1 | 1 |
| *Calochortus_macrocarpus* | 0 | 0 | 0 | 1 | 0 | 0 | 0 | 0 |
| *Carex_geyeri* | 0 | 0 | 0 | 0 | 0 | 0 | 1 | 0 |
| *Chaenactis_douglasii* | 0 | 0 | 0 | 0 | 1 | 0 | 0 | 0 |
| *Chamaebatiaria_millefolium* | 0 | 0 | 0 | 0 | 1 | 0 | 0 | 1 |
| *Chenopodium_leptophyllum* | 0 | 0 | 0 | 0 | 0 | 0 | 0 | 0 |
| *Chrysothamnus_viscidiflorus* | 0 | 0 | 1 | 1 | 1 | 1 | 1 | 0 |
| *Cordylanthus_ramosus* | 1 | 0 | 1 | 0 | 1 | 0 | 0 | 0 |
| *Crepis_acuminata* | 0 | 0 | 1 | 1 | 0 | 0 | 0 | 0 |
| *Delphinium_andersonii* | 1 | 1 | 1 | 1 | 0 | 0 | 0 | 0 |
| *Descurainia_incana* | 0 | 0 | 0 | 0 | 1 | 1 | 1 | 0 |
| *Descurainia_pinnata* | 0 | 0 | 1 | 0 | 0 | 0 | 0 | 0 |
| *Diplacus_nanus* | 1 | 1 | 0 | 0 | 0 | 0 | 0 | 0 |
| *Elymus_elymoides* | 0 | 0 | 1 | 0 | 0 | 0 | 0 | 0 |
| *Eriastrum_sparsiflorum* | 0 | 0 | 0 | 0 | 1 | 0 | 0 | 0 |
| *Ericameria_nauseosa* | 0 | 1 | 1 | 1 | 1 | 0 | 0 | 1 |
| *Erigeron_pumilus* | 0 | 0 | 0 | 1 | 0 | 0 | 0 | 0 |
| *Erigeron_subtrinervis* | 0 | 0 | 0 | 1 | 0 | 0 | 0 | 0 |
| *Eriogonum_caespitosum* | 0 | 0 | 0 | 1 | 0 | 0 | 0 | 0 |
| *Erythranthe_suksdorfii* | 0 | 0 | 0 | 0 | 0 | 0 | 0 | 1 |
| *Festuca_baffinensis* | 0 | 0 | 0 | 0 | 0 | 0 | 0 | 0 |
| *Galium_bifolium* | 0 | 0 | 0 | 0 | 0 | 0 | 0 | 1 |
| *Gayophytum_heterozygum* | 1 | 0 | 0 | 0 | 0 | 0 | 0 | 0 |
| *Hackelia_floribunda* | 0 | 0 | 0 | 0 | 0 | 0 | 0 | 0 |
| *Lappula_redowskii* | 0 | 1 | 0 | 0 | 1 | 1 | 1 | 1 |
| *Lathrocasis_tenerrima* | 0 | 0 | 0 | 0 | 0 | 1 | 1 | 1 |
| *Leymus_cinereus* | 0 | 0 | 1 | 0 | 0 | 0 | 0 | 0 |
| *Lithophragma_tenellum* | 0 | 0 | 0 | 0 | 0 | 1 | 1 | 1 |
| *Lithospermum_ruderale* | 0 | 0 | 0 | 0 | 0 | 0 | 0 | 1 |
| *Lomatium_ambiguum* | 0 | 1 | 0 | 0 | 0 | 0 | 0 | 0 |
| *Lomatium_foeniculaceum* | 0 | 1 | 0 | 0 | 1 | 1 | 1 | 1 |
| *Lomatium_dissectum* | 1 | 0 | 0 | 0 | 1 | 0 | 0 | 1 |
| *Lomatium_idahoense* | 1 | 0 | 0 | 0 | 0 | 1 | 1 | 1 |
| *Mentzelia_albicaulis* | 1 | 1 | 0 | 0 | 0 | 0 | 0 | 0 |
| *Penstemon_cyananthus* | 0 | 0 | 1 | 1 | 0 | 0 | 0 | 0 |
| *Penstemon_procerus* | 0 | 0 | 0 | 0 | 0 | 0 | 1 | 0 |
| *Phacelia_heterophylla* | 1 | 0 | 0 | 0 | 0 | 0 | 0 | 0 |
| *Phlox_aculeata* | 0 | 1 | 0 | 0 | 0 | 0 | 0 | 0 |
| *Phlox_hoodii* | 0 | 0 | 0 | 0 | 1 | 1 | 1 | 1 |
| *Phlox_longifolia* | 1 | 0 | 0 | 1 | 0 | 1 | 1 | 0 |
| *Purshia_tridentata* | 0 | 1 | 1 | 1 | 0 | 1 | 1 | 1 |
| *Ranunculus_glaberrimus* | 0 | 0 | 0 | 0 | 0 | 0 | 0 | 0 |
| *Ribes_aureum* | 0 | 0 | 0 | 0 | 0 | 1 | 1 | 0 |
| *Senecio_triangularis* | 1 | 1 | 0 | 0 | 1 | 1 | 1 | 1 |
| *Sisymbrium_altissimum* | 0 | 0 | 1 | 1 | 0 | 0 | 0 | 0 |
| *Stephanomeria_tenuifolia* | 1 | 1 | 0 | 0 | 0 | 0 | 0 | 0 |
| *Thinopyrum_junceum* | 0 | 0 | 0 | 0 | 0 | 0 | 0 | 0 |
| *Thlaspi_arvense* | 0 | 0 | 0 | 0 | 0 | 0 | 0 | 0 |
| *Toxicoscordion_paniculatum* | 0 | 1 | 1 | 0 | 0 | 0 | 0 | 0 |
| *Viola_glabella* | 1 | 1 | 0 | 0 | 1 | 1 | 1 | 1 |

**Supplemental Table 11.** Model probabilities for the total kipuka plant community and eight separate kipukas using RF and ABC. For this, the reference data was made up of data simulated under OU models of trait evolution and data simulated under BM models of trait evolution.

**Supplemental Table 12.** Model probabilities for environmental filtering for the eight kipuka plant communities using RF and ABC, as well as the median $t_{E}$estimates using ABC.

**Supplemental References**

Butler, M.A. & King, A.A. (2004). Phylogenetic Comparative Analysis: A Modeling Approach for Adaptive Evolution. *Am. Nat.*

Cornwell, W.K., Schwilk, D.W. & Ackerly, D.D. (2006). A trait-based test for habitat filtering: Convex hull volume. *Ecology*.

Garland, T., Harvey, P.H. & Ives, A.R. (1992). Procedures for the analysis of comparative data using phylogenetically independent contrasts. *Syst. Biol.*

Hansen, T.F. (1997). Stabilizing Selection and the Comparative Analysis of Adaptation. *Evolution.* 51, 1341-1351.

Janzen, T., Höhna, S. & Etienne, R.S. (2015). Approximate Bayesian Computation of diversification rates from molecular phylogenies: Introducing a new efficient summary statistic, the nLTT. *Methods Ecol. Evol.*, 6, 566–575.

Kembel, S.W., Cowan, P.D., Helmus, M.R., Cornwell, W.K., Morlon, H., Ackerly, D.D., *et al.* (2010). Picante: R tools for integrating phylogenies and ecology. *Bioinformatics*, 26, 1463–1464.

Kraft, N.J.B. & Ackerly, D.D. (2010). Functional trait and phylogenetic tests of community assembly across spatial scales in an Amazonian forest. *Ecol. Monogr.*

Purvis, A. & Rambaut, A. (1995). Comparative analysis by independent contrasts (CAIC): An apple macintosh application for analysing comparative data. *Bioinformatics*.

Webb, C.O., Ackerly, D.D. & Kembel, S.W. (2008). Phylocom: Software for the analysis of phylogenetic community structure and trait evolution. *Bioinformatics*, 24, 2098–2100.

Webb, C.O., Ackerly, D.D., McPeek, M. a. & Donoghue, M.J. (2002). Phylogenies and Community Ecology. *Annu. Rev. Ecol. Syst.*, 33, 475–505.
